# Towards Dynamic Controller Placement in Software Defined Vehicular Networks

**DOI:** 10.3390/s20061701

**Published:** 2020-03-18

**Authors:** Soufian Toufga, Slim Abdellatif, Hamza Tarik Assouane, Philippe Owezarski, Thierry Villemur

**Affiliations:** 1 LAAS-CNRS, Université de Toulouse, CNRS, 31400 Toulouse, France; eh_assouane@esi.dz (H.T.A.); owe@laas.fr (P.O.); 2 LAAS-CNRS, Université de Toulouse, CNRS, INSA, 31400 Toulouse, France; 3 LAAS-CNRS, Université de Toulouse, CNRS, UT2J, 31100 Toulouse, France; villemur@laas.fr

**Keywords:** vehicular communication, controller placement problem, optimization, SDN, software defined vehicular network

## Abstract

The emerging SDVN (Software Defined Vehicular Network) paradigm promises to bring flexibility and efficient resource utilization to vehicular networks, enabling the emergence of novel Intelligent Transportation Services. However, as it was initially designed with wired network in mind, applying the SDN paradigm to a vehicular context faces new challenges related to the peculiar characteristics of this network (high node mobility and node density, and the presence of wireless links). In this paper, we focus on one of the critical architectural elements of SDVN, namely, the SDN Controller Placement, and promote the use of dynamic placement methods that take into account the dynamicity of vehicular networks’ topology. We also describe the different approaches towards a dynamic controller placement and also propose an ILP (Integer Linear Programming) based dynamic placement method that adaptively readjusts the number and placement of controllers according to road traffic fluctuations. The proposed method is evaluated using a realistic traffic trace from Luxembourg City. Simulation results show that our approach outperforms the static approach as proposed in the literature.

## 1. Introduction

A variety of emerging Intelligent Transportation Systems (ITS) services related to cooperative and automated driving are envisioned for the near future [1,2,3]. Most are expecting from the network strict performance requirements in terms of message transfer delay, reliability and bandwidth requirements [2,4]. The firmness of these requirements makes current wireless technologies unsuitable, and one possible research direction that is gaining adhesion is to consider a Software Defined Network (SDN) based hybrid (LTE (Long Term Evolution) based and DSRC (Dedicated Short Range Communication), etc.) vehicular network as the access network infrastructure to support these emerging services [5,6,7]. Indeed, the ”logical” centralized control based on a thorough visibility of the network, combined with the fine-grained and programmable selection and forwarding treatments of flows, inherent to SDN, can bring a noticeable boost to the emergence of these services. For instance, the selection of the network to be used by a given vehicle at a given time, can take advantage of the global view of the network to make more informed decisions [8]. Furthermore, the ability to reconfigure the network on the fly helps adapting network parameters to a given situation, for example, the transmission power can be adjusted based on the density and mobility of vehicles, in order to mitigate interference and improve network performance [9].

Most existing research work have been elaborated on the opportunities that the SDVN concept could bring when applied to vehicular networks [10]. However, some challenges need to be addressed to reach these expected opportunities. Among these challenges, we mention firstly, the network topology discovery, which is a crucial service in the Software Defined Vehicular Netowrk (SDVN) system [11]. Its main goal is to build and maintain an up-to-date view of the underlying network. The density and high mobility of vehicles make the design of this service a challenging task. Secondly, the connectivity between the vehicles and their controller is partly wireless [12]. In fact, vehicles can go through areas without network coverage, this could make the controller temporarily unreachable, which may impact network performance. Thirdly, the mobility of vehicles can result in frequent changes of their attached SDN controller. This impacts the established sessions with the initial controller, as well as an increase of network overhead due to inter-controller vehicles information exchange. The SDN controller placement is a key architectural element that can help mitigating the above mentioned challenges. It consists of computing the number of SDN controllers to deploy and derive their placement in the network. This choice must be done appropriately given the critical role of an SDN controller in this architecture. First, wireless link connectivity and quality must be considered in order to minimize the connectivity losses between vehicles and their controllers. Second, the SDN Controller coverage should be defined carefully taking into account the density and mobility of vehicles. This helps avoiding SDN controllers’ overload as well as improving the performance of vehicle-to-controller communications, and also, minimizing the number of changes in the set of deployed controllers.

Even if the SDN controller placement problem has been widely covered in the literature, as described in References [13,14], most existing work focused on wired networks and aimed at computing an off-line optimal placement. In this paper, as in Reference [15], the focus is on controller placement in a SDVN context. In comparison to Reference [15], we argue that an online dynamic controller placement strategy that follows road traffic changes or react to some sudden or unexpected road events, should be used in an SDN based vehicular network. More precisely, the contributions of this work are threefold: (1) We assess the performance limitations of a static controller placement on a realistic road traffic model; also (2) we investigate the main alternatives to devise a dynamic controller placement strategy; and finally, (3) we propose extensions to the ILP (Integer Linear Programming) based controller placement method of Reference [15] by including: in the one hand, controllers’ load enabling a fair distribution of vehicles among controllers; and on the other hand, a replacement cost, which, on the advent of a controller replacement decision, guides the selection towards a new placement with a reasonable number of changes in the controllers set. Our performance analysis assesses the gain brought by our proposed dynamic controller placement method in terms of the number of deployed controllers and vehicle-to-controller delays.

This paper is organized as follows. Section 2 presents the Software Defined Vehicular Network architecture. Section 3 gives a description of related works. The main limitations of a static placement are highlighted in Section 4. Section 5 presents the proposed approach, while Section 6 addresses the performance evaluation. Section 7 discusses the obtained results. Finally, Section 8 concludes this paper.

## 2. The Considered Software Defined Vehicular Network Model: Architecture, Motivations and Challenges

In order to support the above cited emerging ITS services, it is envisioned that the communication infrastructure will rely on several emerging paradigms. The cloud computing is a key enabler of ITS services, it is used by the ITS system actors to collect, process and exchange data to provide vehicle with relevant services [16,17]. On the other hand, fog and edge computing are crucial to support ITS services with stringent latency requirements, by bringing processing closer to vehicles [16,18]. In this work, we focus mainly on the network communication component. The scientific community asserts that a multi-RAT (Radio Access Technologies) architecture, combining various access technologies (mainly DSRC and LTE/5G) will be considered to provide the network communication services that meet ITS services’ requirements [1,19]. To this end, an SDN based architecture, combining the two complementary technologies, is proposed in the literature [12,17]. This enables the management of these networks through a unified model (e.g., OpenFlow-like protocol), also allows optimizing network resources utilization, thanks to the programmability and global vision provided by SDN. These architectures are known as SDVN (Software Defined Vehicular Network).

**Figure 1:**
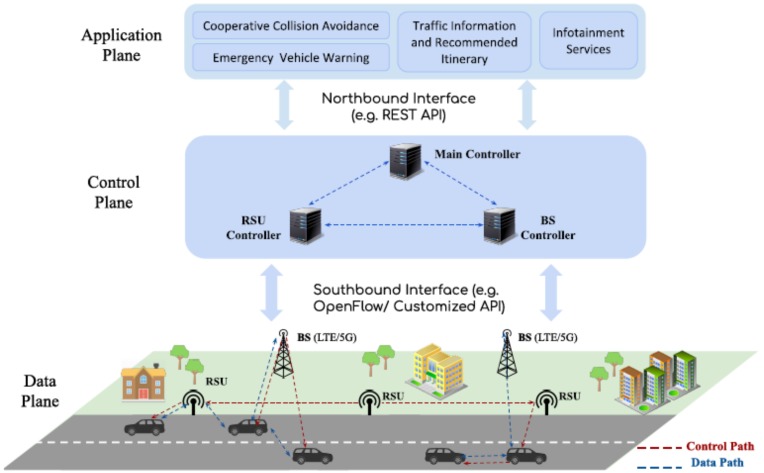
Conceptual view of Software Defined Vehicular Network (SDVN) architecture.

Figure 1 depicts a conceptual view of an SDVN architecture, organized in three planes, data, control and application planes [17]. The data plane is composed of heterogeneous forwarding elements—static nodes—BSs (Base Station) and RSUs (Road Side Unit) communicating with 5G/LTE and DSRC, respectively, and mobile nodes (vehicles), equipped with both LTE and DSRC interfaces. We assume that all these nodes (BS, RSU, Vehicle) are programmable via SDN (e.g., Openflow/customized Southbound Interface). We consider a two-level hierarchical control plane, composed of local controllers that manage BSs and RSUs (level 1 controllers), using the information sent by the forwarding elements, and a global controller (level 2 controller) used to coordinate lower level controllers. Based on the information exposed by level 1 controllers, the main controller is able to jointly control these networks (i.e., LTE and DSRC) in a scalable way.

## 3. Controller Placement in SDVN: Problem Definition and Related Work

In this section, we present the controller placement problem, we firstly discuss the related work in wired networks, then we focus on the case of SDVN. Precisely, we highlight the characteristics of SDVN that impose new challenges to the controller placement problem. Then, we present the corresponding related work and we describe our contributions.

### 3.1. Controller Placement in SDN

In an SDN architecture, network intelligence becomes centralized in SDN controllers, which implies that the controllers communicate continuously with lots of forwarding nodes, on the one hand, to obtain network state information, and on the other hand, to apply various network policies (e.g., Forwarding rules). These continuous exchanges should not affect the overall performance of the network. Therefore, It is clear that the placement of the controller with respect to forwarding nodes is a crucial element to consider in these architectures. The impact of SDN Controller placement on network performance was initially studied by Heller et al. [20] in wired networks. It has been shown that the optimal placement can reduce the average latency by a ratio of up to 5, compared to a random choice. In addition, the number of controllers and their placement depend on the network topology and the desired reaction bounds. Furthermore, this problem attracted more attention in the distributed control plane architecture, proposed in order to address scalability and robustness issues in SDN networks. The goal of the proposed approaches is to find the best placement strategy (i.e., controller placement in the network and switch-controller mapping) that optimizes one or more performance metrics. Latency between controllers and switches represents the main considered metric by the majority of work in the literature, some additional metrics were introduced such as, capacity and load balancing, inter-controller communication delay, deployment cost and energy consumption.

The problem is often formalized as a Facility Location Problem. Clustering methods were investigated to solve the problem. The authors of Reference [21] propose a modified K-means method to minimize latency between controllers and nodes. The results show that the proposed algorithm can reduce the maximum latency by a ratio of 2.4 compared to standard K-means. The authors of Reference [22] analyze the trade-off between latency and load balancing. Their proposed approach relies on a Linear Programming formulation. The results show that the control plane load can be well balanced with a limited increment on the delay. Similarly, the work in Reference [23] proposes to use Bargaining Game in order to find a trade-off between switch-controller latency, inter-controller latency, and load balancing between controllers. Simulation results show that their proposed approach ensures a better trade-off compared to a mono-objective approach. Multi-objective optimization was also considered by Reference [24], in this work, the authors proposed a Pareto-based Optimal COntroller placement (POCO) with a focus on the resilience of controllers. They tried to minimize latency and load balancing in case of controller failures. Their finding affirmed that for most of the considered topologies, more than 20% of all nodes should be controllers to ensure a continuous connection of all nodes to one of the controllers in any arbitrary double link or node failure scenario. An extension of Reference [24] based on a heuristic approach is proposed in order to avoid computational overhead [25].

All the above cited works focus on static placement, which means that the placement is computed once offline and deployed on the network. If network traffic load evolves over time, the initial placement may no longer guarantees the expected performance. To overcome this problem, some proposed approaches try to readjust switch-controller mapping according to traffic dynamics, that is, migrating switches from one controller to another. The challenge is to select the most appropriate switch to migrate to the relevant controller. In Reference [26], authors propose a switch migration approach in order to balance controllers load. Simulation results show that their proposed approach reduces the load disparity between SDN controllers by 40% by migrating only a small number of switches. In addition to switch migration, the authors of Reference [27] propose to adjust the number of active controllers (from a predefined set) while ensuring minimal flow setup time (i.e., a controller is considered active if at least one switch is assigned to it, otherwise, it is considered inactive). The simulation results show that the proposed framework successfully provisions SDN controllers based on traffic fluctuations. Similarly, the authors of Reference [28] proposed a non-zero-sum game based approach in order to minimize the number of active controllers with bounds on controller loads and latency. This approach guarantees the maximum utilization of SDN controllers but did not determine the controller placement on the network. Considering both the traffic load and latency constraints, Huque et al. [29] propose an approach to determine firstly the locations of controller modules (set of controllers) to bound communication latencies, then, the number of controllers per module to support the dynamicity of network load. Simulation results show that the proposed approach maximizes the utilization of SDN controllers under latency constraints. The adaptive mentioned approaches are event-based. Capacity is used in References [26,28,29] and flow setup time in Reference [27]. More precisely, in Reference [26] the switch migration is triggered once the controller load exceeds a predefined threshold. While, in Reference [27], the proposed algorithm is executed periodically at each interval T (1 h), using input metrics calculated on the previous slot (e.g., Flow Setup Time). No details about frequency of iterations were provided in References [28,29]. However, the impact of the timestamp of migration (interval T) as well as the migration cost were not analyzed.

### 3.2. Controller Placement in SDVN

#### 3.2.1. Problem Description and Motivating Preliminary Analysis

Taking into account the requirements of critical ITS applications, we consider that the controllers are co-located with RSU entities. This makes the controllers closer to the vehicles, which reduces significantly the controller-node latency compared to a placement in the cloud [15]. In addition, there is no need for an additional devoted infrastructure to host SDN Controllers. The controller placement problem in this case aims at finding the optimal set of RSU to be designated as controller, and the mapping between RSU entities and the chosen controllers, with the purpose of optimizing a given metric(s) (e.g., latency).

The density and high mobility of vehicles are the main characteristics that pose new challenges to the controller placement problem in SDVN. Firstly, RSU entities have limited resources (i.e., computation, storage), which limits their ability to manage a large number of nodes, compared to SDN controllers located in data-centres. Secondly, in our considered SDVN model, the vehicles are programmable via SDN, hence, the link quality between a programmable vehicle and its controller (chosen RSU) is not guaranteed, especially if the vehicle is not directly under the coverage of its RSU, and multi-hop paths to the vehicle are needed. In addition, the network topology becomes highly dynamic, with high spatio-temporal variations, notably, in urban scenarios. Therefore, the vehicles may change the controllers frequently, which increases inter-controllers synchronisation overhead. To minimize these changes, one direction is to consider a placement of controllers with a large coverage (in terms of controlled RSUs). However, this leads in increasing the controller load (in number of managed nodes (RSU, Vehicle)), as well as the distance between controllers and nodes (in number of hops). Both contribute to increase the controller-node latency.

**Figure 2:**
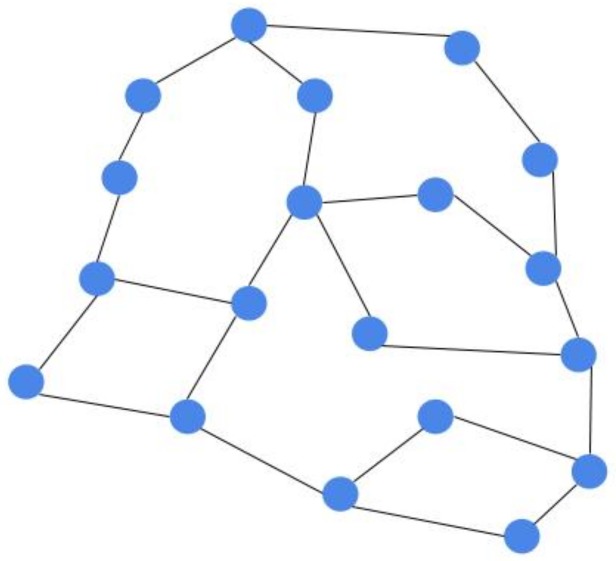
Road topology used, Road Side Units distributed uniformly.

In order to show the impact of controller coverage (number of hops between a controller and its associated nodes) on network performance, we use the Mininet-WiFi emulator [30], and we consider the road topology, used in Reference [15], and shown on Figure 2. Each node represents an OpenFlow RSU, and one RSU is designated as controller with an In-band control mode. Three scenarios are considered with different number of vehicles (20, 50, 100). We change the controller placement for each scenario by increasing the number of hops between controller and vehicles from 2 to 6 with a step of 1.

Two metrics are considered in our evaluations:

- Flow Setup Time: represents the difference between the time a node (RSU/Vehicle) sent a Packet-In to the controller and received its corresponding Packet-Out response message.
- Overhead: represents the total number of Packet-In messages generated by all the nodes (RSU/Vehicle).

**Figure 3:**
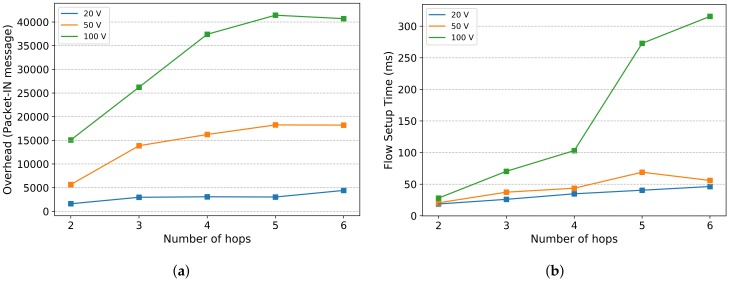
Placement performance. (**a**) Overhead; (**b**) Flow setup time.

Figure 3a,b show respectively, the overhead and the average flow setup time as a function of the number of hops and the number of vehicles.

We can notice that both delay and overhead increase as a function of the number of hops between the controller and the vehicles, for the three considered scenarios. This increase is more significant in the case of 100 vehicles. Therefore, a placement strategy that aims to minimize the node-controller latency, should consider that the majority of the controlled nodes (vehicles) are close to their controller, and preferably, a reduced number of controlled nodes per controller.

#### 3.2.2. Related Work

Recently, the placement problem has been investigated in other network domains, such as wireless networks [31,32], cellular networks [33] and vehicular networks [15]. The problem is different from the wired network case, especially when the considered south-bound interface is wireless. The quality of wireless links is a key element in determining the subset of links to activate [31]. The user location is also crucial in deriving the eNodeB request rate in cellular networks [33].

In this work, we focus on the case of SDVN. Liyanage et al. propose an initial study of Controller Placement Problem on SDVN [15]. They focus on minimizing controller-node latency. Their proposed approach privileges to place controllers at RSU with strategic location (e.g., intersection with higher load), in order to ensure that a maximum number of vehicles are close to their controllers for a decent period. Results show that placing controllers at RSU level reduces latency compared to a placement in the cloud. Although, the optimal solution shows lower delays compared to a random placement of controllers.

However, with road traffic fluctuations, this static placement may no longer be efficient, for example, a traffic jam in a given area may result in a high number of vehicles located far from the deployed controllers. A detailed analysis of the weaknesses of a static placement on SDVN is presented in Section 4.

To overcome this problem, we explore the dynamic placement of controllers in SDVN. The main idea is to adapt controller placement based on road traffic changes. Our analyses are based on realistic traffic scenario compared to the simplified small network used in Reference [15].

Table 1 summarizes our positioning with respect to related work.

## 4. Main Limitations of Static Placement in SDVN

In order to show the shortcomings of a static placement in a SDVN context, we use a realistic mobility trace to analyze the impact of traffic changes on the performance of the placement. Firstly, we illustrate these impacts, and then we present the used setup and the considered mobility scenario. Finally, we review the main results.

**Figure 4:**
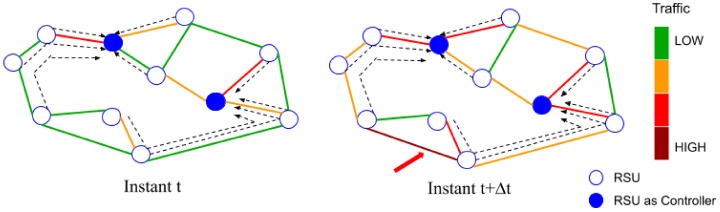
Simplified representation of traffic changes during the day.

Figure 4 illustrates a simplified road network, represented as a graph. Each node represents an RSU entity. The links represent the roads, each link is colored according to the traffic density in each road. Blue nodes represent deployed controllers, and dotted lines represent the node-controller mapping. The traffic evolution during a day implies variations on the number of vehicles and the vehicle density distribution. For example, the roads and places solicited during the business days are different from the week-end ones, as shown in the traffic analysis in Reference [34].

These variations mainly affect two factors, namely: (i) the controller load (in number of controlled nodes (vehicles)), and (ii) the number of nodes being far from their controller, as shown in right graph. The instantiated controllers become overloaded (increase in number of vehicles) and several nodes are located at 3 hops from their controllers (area pointed with red arrow). This results in an increase of controller-node latency, as explained in the previous section. The following simulations analyze these aspects using a realistic traffic scenario.

### 4.1. Experimental Setup

**Figure 5:**
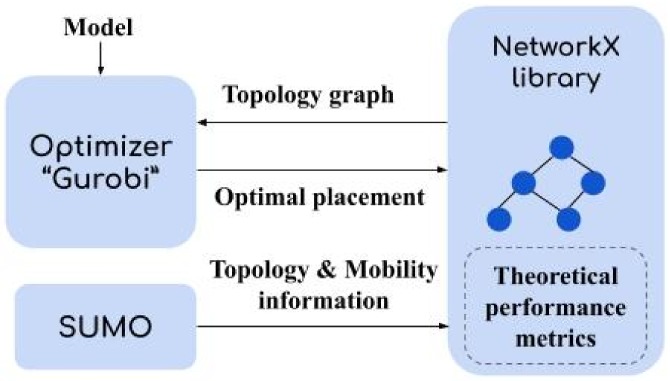
Used setup based on theoretical analysis.

In our analysis, we consider a realistic mobility scenario (described below) with a large number of nodes (RSU, vehicles). Mininet-wifi suffers from scalability issues as the number of vehicles per simulation is no more than 100. In addition, the interface between SUMO & Mininet-wifi is still under development, preventing the use of realistic mobility models. This is why, our setup is in fact composed of three components: NetworkX (graph library) [35] combined with mobility and traffic simulator SUMO [36], and Gurobi solver [37] to implement optimization models. Figure 5 depicts the combination of these tools.

Based on the findings of the experiments of Figure 3, we define three graph-based metrics: Controller load, Number of vehicles per hop and Average hop.

- Controller load: represents the number of nodes (RSU, Vehicles) managed by a given controller at a given time;
- Number of vehicles per hop: Number of vehicles located at a given hop from their controller, at a given time;
- Average hop: Average distance (in number of hops) between the nodes and their controllers at a given time.

### 4.2. Mobility Scenario-LuST

**Figure 6:**
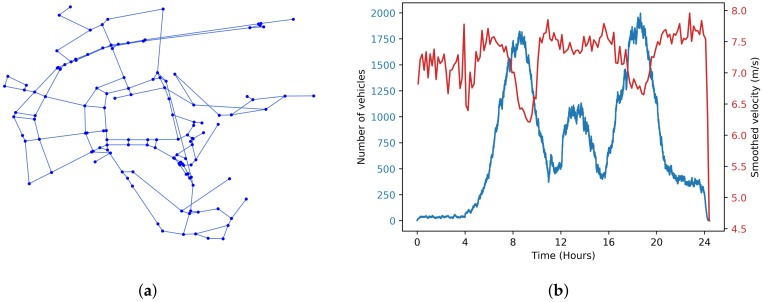
Considered Mobility Scenario. (**a**) Extracted graph; (**b**) Mobility information.

We consider the Luxembourg SUMO Traffic (LuST) Scenario by Codeca et al. [38]. It is generated using SUMO and is realised in Luxembourg city. The trace reproduces the mobility behavior of almost 300,000 vehicles composed of different types of vehicles (personal vehicles, public transport vehicles, etc.) in an area of 156 Km${}^{2}$, during 24 h. In our study, we focus mainly on the urban scenario; for instance, we select an area of 2 × 2 Km${}^{2}$, in the city-center. We assume that an RSU entity is located at each intersection. Figure 6a shows the extracted road topology (converted on graph, used thereafter as input by the model). The evolution of number of vehicles and their average velocity in this selected area is shown in Figure 6b.

### 4.3. Simulation Results

The goal of the following simulations is to show the impact of traffic variations on the efficiency and performance of the controller placement strategy, using the metrics described above. We implemented the model proposed in Reference [15] (presented in Section 3.2), with a maximum controller coverage of 3 hops, and RSU coefficients calculated using the average load of the day, as specified by the authors.

**Figure 7:**
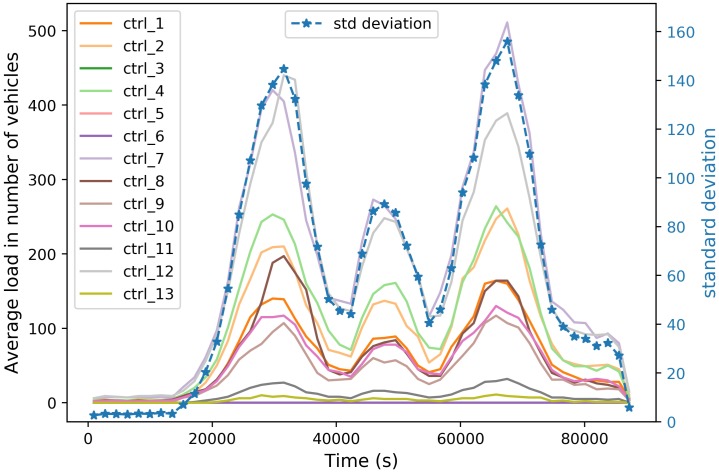
Average load per controller.

Figure 7 shows the evolution of the controllers’ load (number of controlled vehicles) during the day. We can notice that the controllers’ load (presented on left *y*-axis) follows the same trend as the evolution of the number of vehicles during the day (presented on Figure 6b). The most overloaded controller reaches a maximum overload of 500 Vehicles (∼25% of instantiated vehicles at this time) which impacts network performance, as explained in the previous section. We also notice that there is a significant difference between controllers in terms of load, as highlighted on right *y*-axis (standard deviation); this can be explained by the difference on traffic demand at each area, during the day.

**Figure 8:**
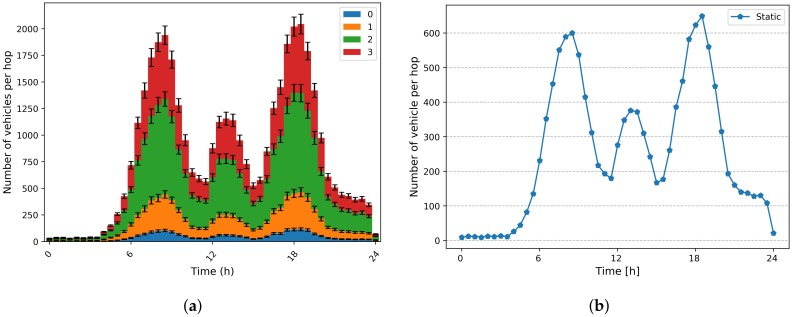
Placement performance. (**a**) Number of vehicles per hop; (**b**) Number of vehicle per 3 hop.

Mobility also impacts the density of vehicles [number of vehicles/km${}^{2}$] in a given area and at a given moment, which mainly affects the distance between vehicles and their controllers (distance in number of hops). Figure 8a shows the number of vehicles at a given distance (1,2 and 3 hops) from their controller. We can notice that the number of vehicles situated at a distance of 3 hops increases during the rush hours, around 600 vehicles (∼30 % of instantiated vehicles at that time) as highlighted on Figure 8b. This impacts network performance as explained above.

It is noticeable that a static placement strategy is not adapted for the SDVN context, and that a dynamic placement according to the vehicles’ mobility seems more relevant.

## 5. Proposed Model

### 5.1. Notations

The RSU network is modeled as a Graph G=(V,E), where *V* is the set of RSU nodes and *E* the set of links connecting these nodes.

We denote the distance matrix *D*, where $d_{ij}\in D$ represents the distance from $i^{th}$ RSU to $j^{th}$ RSU in terms of number of hops. The term m=|V| denotes the total number of RSU and the term *L* represents the number of RSU nodes designated as an SDN controller.

The controller coverage (in number of hops) is denoted as *S*, which represents the maximum distance in number of hops between a given RSU and its controller. The term $z_{j}$ represents The total number of managed nodes (RSU, vehicles) by a controller *j*.

The binary variable $x_{ij}$ is used to represent the selected RSU controllers. More precisely, $x_{ij}=1$, if RSU *i* is attached to controller *j*, otherwise $x_{ij}=0$.

In order to favor the placement of controllers on RSUs that sit in a strategic location and that have high regular traffic, as proposed in Reference [15], a coefficient $C_{i}=\left\{C_{1},C_{2},\ldots ,C_{m}\right\}$ is assigned to each RSU, based on its location and mobility characteristics of the area it covers. It is calculated as follows: $C_{i}=p*\frac{R_{n}*V_{r}}{V_{s}}$, with *p* a negative constant, $R_{n}$ the degree of RSU node (i.e., Number of direct neighboring RSU), $V_{r}$ the average vehicle passing rate, and $V_{s}$ the average vehicle speed. Table 2 summarizes the used notations.

### 5.2. Objective Function

#### 5.2.1. Node-Controller Latency

One of the main objectives of placement is to minimize the latency between the node and its controller. In our model, the latency is represented by the distance (in number of hops) between the node and its controller (denoted by $d_{ij}$). The maximum distance between a node and its controller is bounded by the controller coverage *S*. Constraint (1) is introduced in the model in order to guarantee that the maximum distance between a vehicle (directly under RSU coverage) and its controller does not exceed the specified controller coverage.

(1) $$d_{ij}\cdot x_{ij}\leq S-1.$$

To minimize the latency, we added the Equation (2) to the objective function, which tries to minimize the distance between the controllers and their attached nodes.

(2) $$\sum_{i=1}^{m}\sum_{j=1}^{m}\left(S-d_{ij}\right)C_{i}x_{ij}.$$

Here the $C_{i}$ coefficient is used to privilege the RSUs with a strategic location as specified initially in Reference [15]. The term *S* denotes the controller coverage, $d_{ij}$ the distance in number of hops from RSU *i* to RSU *j*, and $x_{ij}$ the node-controller mapping, as explained above.

#### 5.2.2. Load Balancing

Given the limited resources of an RSU node (processing capacity, memory). The selected RSU (as controller) can only manage a limited number of nodes. One of the objectives of the model is to balance the load between the various selected controllers. We define $z_{j}$ the controller load in terms of number of vehicles, it is computed as follows:

(3) $$z_{j}=\sum_{i=1}^{m}x_{ij}\times NoV\left(i\right)$$

with NoV(i) being the number of covered vehicles by RSU *i*, and $x_{ij}$ the node-controller mapping, which represents the set of attached RSU to Controller *j*.

The idea is to compute the difference in the number of vehicles that each controller manages. It is computed as follows:

(4) $$\beta \sum_{j=1}^{m}\sum_{j^{\prime }=1}^{m}{\left(z_{j}-z_{j^{\prime }}\right)}^{2}.$$

The Equation (4) can be linearized with a simplified heuristic as follows:

(5) $$\beta (z_{max}-z_{min})$$

with

(6) $$\begin{array}{c} \forall z\in Z,z_{min}\leq z\leq z_{max}. \end{array}$$

This Equation (5) is added to the objective function. β is the load balancing coefficient. $z_{min}$ and $z_{max}$ represent, respectively, the lowest and highest controller load.

#### 5.2.3. Replacement Cost

In our approach, we aim to adapt the controller placement according to the road traffic evolution. This means that some controllers may be added and/or removed, which implies a change of controller-node mapping. These changes increase synchronization overhead between controllers. We would like to minimize these changes while adapting to the new traffic conditions.

Let’s denote as $\left\{{\hat{x}}_{ij}|i,j\in \left[1,m\right]\right\}$, the last computed placement. When road conditions change requiring a controller replacement, one possible objective is to favor the placement that do not induce significant changes to the current controller placement.

This is expressed by minimizing the difference between $\left\{x_{ij}|i,j\in \left[1,m\right]\right\}$ and $\left\{{\hat{x}}_{ij}|i,j\in \left[1,m\right]\right\}$.

(7) $$\gamma \sum_{i=1}^{m}\sum_{j=1}^{m}\left(x_{ij}\times \left(1-{\hat{x}}_{ij}\right)+{\hat{x}}_{ij}\times \left(1-x_{ij}\right)\right).$$

Note that $x_{ij}$ is a binary variable (node-controller mapping) and ${\hat{x}}_{ij}$ is a binary constant (last computed placement). The Equation (7) is added to the objective function. γ is the replacement coefficient. We could give a large value to this coefficient so that changes to current state can be minimized.

#### 5.2.4. Number of Controllers

One of the classic objectives in the controller placement problem, is to reduce the number of deployed controllers, mainly, for cost and maintenance reasons. The number of controllers is computed as follows:

(8) $$L=\sum_{j=1}^{m}x_{jj},$$

With $x_{jj}$ that represents if RSU *j* is a controller or not (1 if controller, 0 otherwise)

### 5.3. Constraints

Equation (9) expresses the fact that, at a given time, a node can be controlled by only one controller.

(9) $$\forall i:\sum_{j}^{m}x_{ij}=1.$$

The global model can be summarized as follows:

(10) $$\begin{array}{cc} \mathrm{minimize}\;\; & \alpha .L+\sum_{i=1}^{m}\sum_{j=1}^{m}\left(S-d_{ij}\right)C_{i}x_{ij}\\ \; & +\beta (z_{max}-z_{min})\\ \; & +\gamma \sum_{i=1}^{m}\sum_{j=1}^{m}\left(x_{ij}\times \left(1-{\hat{x}}_{ij}\right)+{\hat{x}}_{ij}\times \left(1-x_{ij}\right)\right), \end{array}$$

with

(11) $$\begin{array}{c} \forall z\in Z:z_{min}\leq z\leq z_{max} \end{array}$$

(12) $$\forall j:z_{j}=\sum_{i=1}^{m}x_{ij}\times NoV\left(i\right)$$

(13) $$\forall i,j:d_{ij}\cdot x_{ij}\leq S-1$$

(14) $$\forall i:\sum_{j}^{m}x_{ij}=1$$

(15) $$L=\sum_{j=1}^{m}x_{jj}$$

(16) $$\forall i,j:x_{ij}=0,1$$

(17) $$L,z_{j}\in Z^{+}.$$

## 6. Performance Evaluation

We used the same setup and mobility scenario described in Section 4. The proposed ILP model (10) is implemented using Gurobi Solver, enriched with mobility information from the LuST scenario. The goal is to show the benefits of an adaptive controller placement approach in a SDVN context. We focus mainly on the node-controller latency (captured using *number of vehicles per hop* metric). Two scenarios were considered, *Dynamic-I* and *Dynamic-II* with and without cost of replacement, respectively. In both scenarios, the placement is computed at regular intervals (every 4 h) during the day, with updated information about mobility.

### 6.1. Dynamic I—Placement without Replacement Cost

**Figure 9:**
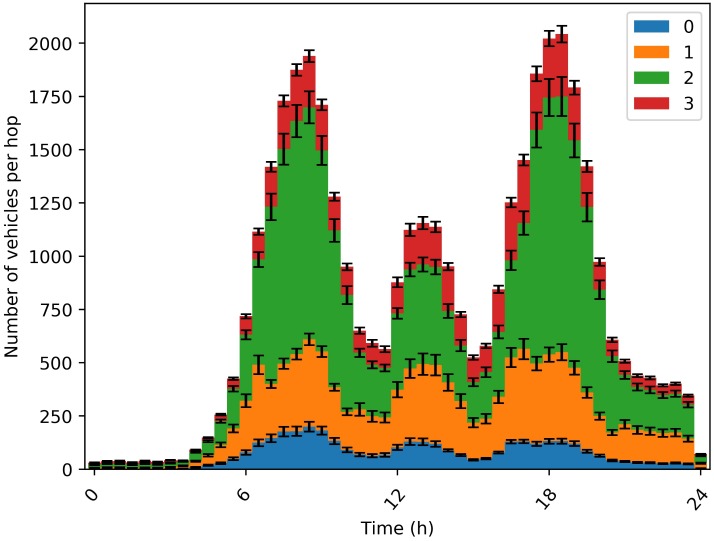
Number of vehicles per hop—Dynamic-1.

Figure 9 shows the total number of vehicles located at a given distance from their controller (number of hops between controller and vehicle), during the day. We can notice that the majority of vehicles are close to their controller (less than 3 hops), even at rush hours, compared to a static placement (previously presented in Figure 8a).

**Figure 10:**
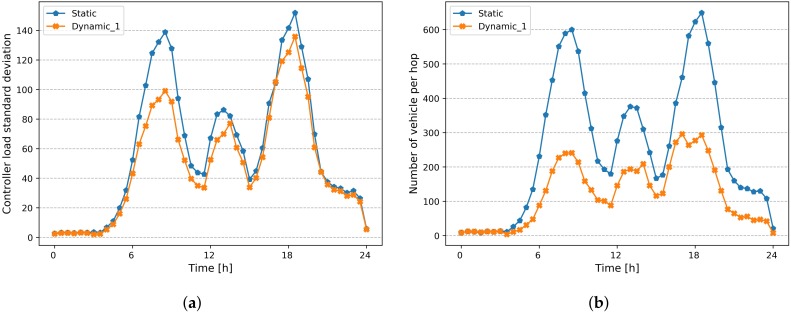
Comparison of Static and dynamic Placement strategies. (**a**) Standard deviation of Controller Load; (**b**) Number of vehicle per 3 hops.

In order to show the benefits of a dynamic placement strategy, we compare it with the static placement described above. Figure 10a,b show respectively, the standard deviation of controller load and number of vehicles located at 3 hops, for both static and dynamic placement strategies. We can notice that the number of vehicles at 3 hops decreases from an average around 600 to 250 vehicles, during rush hours, thanks to the readjustment of controller placement. Furthermore, adding new controllers results in a reduction of the standard deviation of controllers’ load, as shown in Figure 10a.

**Figure 11:**
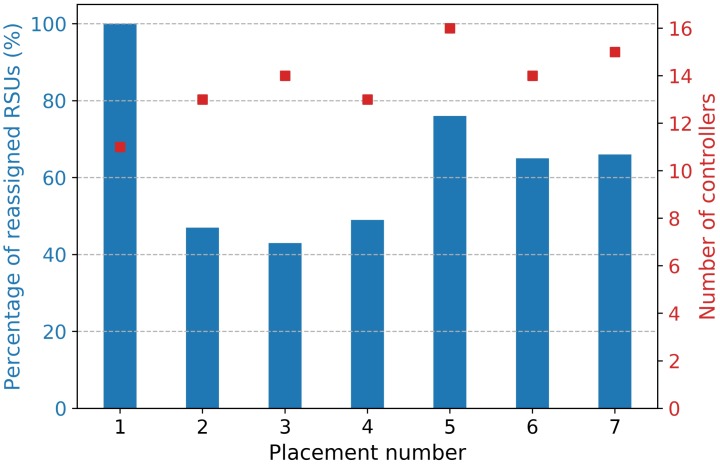
Percentage of reassigned RSUs.

The gain brought by the dynamic approach compared to the static strategy is due to an adjustment of the number and placement of the controllers during the day. In the static placement case, the number of controllers is fixed (13 controllers), as presented in Figure 7. Whereas, in the dynamic approach, the number of controllers varies from 11 to 16 depending on the time of the day, as shown in Figure 11 (right red *y*-axis). These changes generate a network overhead, as explained in Section 5.2.3.

To quantify the impact of controller replacement on network performance, we defined an additional metric to measure the number of affected nodes by a placement change at a given time instant. Figure 11 (left blue *y*-axis) shows the percentage of affected nodes at each placement. We can notice that a significant number of RSU nodes is affected at each new replacement.

### 6.2. Dynamic II—Placement with Replacement Cost

In this second scenario, we try to minimize the impact of placement modification (pointed out in *Dynamic-I* scenario). Thus, we integrate the *replacement cost* into to the model, in order to minimize these changes, while adapting to traffic changes.

**Figure 12:**
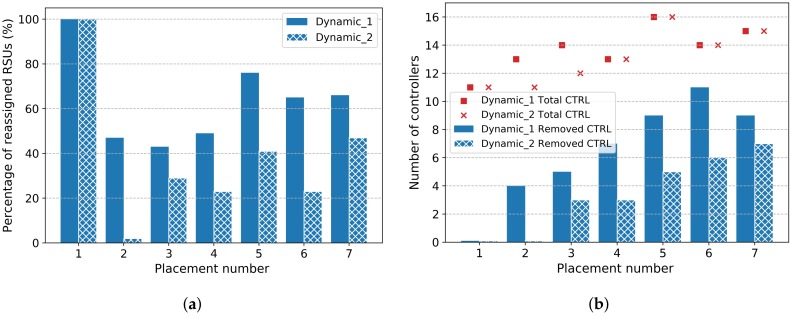
Minimize replacement variations. (**a**) Percentage of reassigned RSUs; (**b**) Number of relocated controllers.

We executed the simulation with the same model parameters of *Dynamic-I* scenario. Further, we integrate the *replacement cost* in the objective function of Equation (10) with a lower coefficient γ. Figure 12a shows the percentage of reassigned nodes at each new replacement, for both *Dynamic-I* and *Dynamic-II* scenarios. We can notice that in *Dynamic-II* scenario (with replacement cost), the number of changes has significantly decreased compared to the *dynamic-I* case. For example, the percentage of changes during placement 2 dropped from 47% to 0%, and a decrease of more than half of changes for placements (4, 5, 6).

In addition, we analyzed the evolution of the number of controllers during the day for both scenarios *(Dynamic-I and Dynamic-II)*. We notice that the number of controllers evolves almost in the same way for both scenarios, except for placement 2 and 3 (two controllers less), as shown in Figure 12b. We also notice that the number of replaced controllers is significantly reduced in *Dynamic-II* scenario, compared to *Dynamic-I*. This greatly reduces the controller-to-controller synchronization overhead.

**Figure 13:**
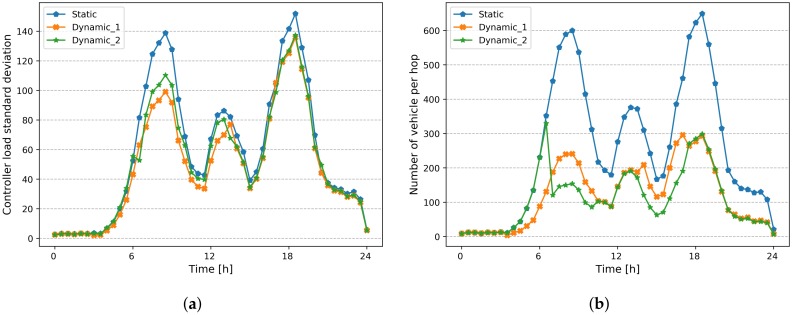
Comparison of Static and dynamic Placement strategies. (**a**) Standard deviation of Controller Load; (**b**) Number of Vehicle at 3 hop.

Moreover, we analyze the impact of reducing the replacement changes on the model performance with regard to the adaptability to road traffic changes. Figure 13a,b show respectively, the standard deviation of controller load and the number of vehicles located at 3 hops, for both scenarios *(Dynamic-I and Dynamic-II)*, compared to static placement strategy. We can notice that the number of vehicles at 3 hops evolves approximately in the same way for both scenarios. The same trend for standard deviation of controller load, which means that we can achieve the same performance with a considerable reduction in the number of changes (i.e., network overhead), by integrating the *replacement cost* objective to the model.

It is clear that minimizing the replacement changes reduces the possibilities of adapting to the new traffic conditions and penalize the other objectives, especially the node-to-controller latency. The γ coefficient can be adjusted appropriately to find a good trade-off between both objectives.

## 7. Discussion

With the emergence of the SDVN architecture, where vehicles represent a programmable network node, the placement of controllers needs to cope with new critical challenges, notably the dynamics of the topology, due to the spatio-temporal variations of the number and density of vehicles.

**Figure 14:**
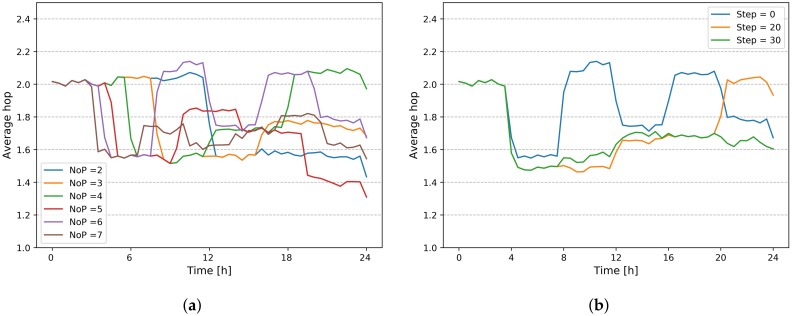
Impact of dynamic parameters on model performance. (**a**) Impact of period T on model performance; (**b**) Benefit of estimated view (Model with NoP = 6).

In our work, we propose an adaptive approach whose purpose is readjusting the placement of controllers according to the road traffic evolution. In order to show the relevance of the proposed approach, we used a periodic placement. However, the choice of the time-period (T) is not obvious and depends on the mobility variations during the day. A large T may not be relevant if significant variations occur between the two placements, while a small T with regular placements can lead to computational overhead, and sometimes a placement with few modifications with respect to the existing placement (e.g., in case of few traffic changes). Figure 14a shows the model performance in terms of average hops, with various T values (i.e., number of placement *NoP*, during 24 h). We can notice the difference between different values of T, which depends of the moment of the replacement in each case.

An event-driven approach seems more relevant in order to trigger replacements. For example, thresholds can be pre-established based on controller load, or the number of nodes being far from their controllers. These changes are mainly triggered by a change in road traffic conditions. Two main limitations arise in this approach. Firstly, the number of events can be very large. This induces regular placement changes which can result in transient placements. Secondly, the duration of an event, can lead to unnecessary changes in controller placement, especially for events with a short duration. For example, an isolated traffic jam can cause the increase in the number of vehicles in a given area.

To overcome this problem, we can enrich the event based approach with an estimated view of road traffic evolution, in order to trigger a replacement in a more appropriate way. Figure 14b shows the benefit of an estimated view (Step = 20, 30) on the placement performance, compared to an instantaneous placement (Step = 0). *Step* represents the length of the estimated view in minutes.

In both approaches (Periodic or Event-based), if recurrent replacements are considered (i.e., a short T value in the periodic approach, or event based with regular traffic changes), it is more relevant to compute the replacement with a higher coefficient weight of the *Replacement-Cost* objective. Thus, the placement adjustment does not generate a significant network overhead.

## 8. Conclusions and Future Work

In this work, we explored the adaptive controller placement problem in a SDVN context. Firstly, we analyzed the main characteristics of vehicular networks that pose new challenges to the controller placement. Based on a realistic trace of mobility from Luxembourg City, we highlighted the weaknesses of a static approach in this context. Then, we demonstrated the benefits of a dynamic approach to adapt to traffic fluctuations during the day.

We evaluated the impact of an adaptive controller placement strategy on network performance and we introduced the *Replacement cost* objective, in order to reduce these changes. Simulation results show that we can achieve a good performance while reducing network changes.

Finally, we discussed the difficulty of choosing a relevant dynamic replacement strategy, and we showed how an approach guided by an estimated view on traffic conditions, can help triggering replacement, effectively.

One direction of our future work is to investigate a placement guided by a machine learning based prediction of road traffic. Secondly, we plan to investigate more placement metrics in order to meet the various reported challenges of controller placement in a SDVN context, more particularly, minimizing inter-controllers synchronization overhead. One direction is to assign RSU entities with highest traffic transitions to the same controller. Finally, we plan to improve our performance analysis with other mobility traces.

## Figures and Tables

**Table 1:** Positioning w.r.t. related work.

Ref	Context	Objective				Approach	
		*Latency*	*Capacity*	*Reliability*	*Co-Re*	*Static*	*Dynamic*
[20]	WAN	✓				✓	
[21]	WAN	✓				✓	
[22]	WAN	✓	✓			✓	
[23]	WAN	✓	✓			✓	
[24]	WAN	✓	✓	✓		✓	
[26]	WAN		✓				✓
[28]	WAN	✓	✓				✓
[27]	WAN	✓	✓		✓		✓
[29]	WAN	✓	✓				✓
[32]	Wireless Nets	✓		✓			✓
[33]	LTE/Mobile nodes	✓	✓			✓	✓
[15]	Vehicular/Dynamic Topo	✓	✓			✓	
**Our Work**	Vehicular/Dynamic Topo	✓	✓		✓		✓

**Table 2:** Notations.

Notation	Definition
*D*	Distance matrix
$d_{ij}$	Distance from $i^{th}$ RSU to $j^{th}$ RSU in terms of number of hops
*m*	Number of RSU node
*L*	Number of RSU node, selected as a Controller
NoV(i)	Number of covered vehicle by RSU *i*
$x_{ij}$	Binary variable (1 if RSU *i* is attached to Controller *j*, otherwise 0)
${\hat{x}}_{ij}$	Binary constant (last computed placement)
$C_{i}$	Coefficient of RSU *i*
*S*	Controller coverage in number of hops
$z_{j}$	Load of controller *j* ( in terms of controlled RSU and Vehicles)
$z_{min}$	The lowest controller load
$z_{max}$	The highest controller load
α,β,γ	Coefficients of model objectives
